# Editorial: Consumer Engagement in Health and Well-being: Theoretical and Empirical Perspectives in Patient Centered Medicine

**DOI:** 10.3389/fpsyg.2017.01811

**Published:** 2017-10-20

**Authors:** Guendalina Graffigna, Elena Vegni

**Affiliations:** ^1^Department of Psychology, Università Cattolica del Sacro Cuore, Milan, Italy; ^2^Department of Health Sciences, University of Milan, Milan, Italy

**Keywords:** consumer engagement, health, well-being, patient-centered medicine, health behavior

The growing understanding of the key role of people in improving healthy behaviors and clinical outcomes has led healthcare to search for innovative ways to foster individuals' roles in the care and health promotion processes. It comes as no surprise, therefore, that making consumers active agents in their health and care is up today recognized as a key priority for services' innovation. In the era of participatory health, the concept of “engagement” may be particularly promising to give consumers a starring role in managing their health and well-being.

The healthcare field has recently introduced the term “engagement” in its lexicon to call for a renewed partnership among the actors (i.e., patients, caregivers, practitioners, decision makers…) implied in the health and care management. Overall, the concept of engagement attempts to offer a compass for action in the current complex and uncertain context of healthcare design and health promotion initiatives. The main aim is giving (back) a leading role to patients and taking them on board for a more efficient and effective process of care delivery and of health promotion initiatives. Furthermore, consumer health engagement can be the key to systematically diagnose and make sense of the different organizational, relational, and psychological components in play in the dynamic exchange between “demand” and “supply” of health and care. This challenge could or even should be integrated with a complex attempt coming from the literature on medicine and regarding a new medical model that should be offered to patients/clients/consumers: that of a patient centered medicine, based on a biopsychological epistemology.

Patient engagement may be defined as an umbrella term that qualifies the systemic relation that occurs between the demand and the supply of healthcare, at different levels and in different situation. If considered according to this meaning, patient engagement overarches the other terms more traditionally used to denote the active role of patients in their care such as patient adherence, patient compliance, patient involvement, patient participation, and patient activation (Graffigna et al., 2016).

Precisely, the concept of “activation” differs from the concept of “engagement” since it is mainly referred to the knowledge, attitude, and skills of patients in their self-management; while the concept of “engagement” is also applicable to the patients' proactive role in other settings, such as health promotion and prevention. Furthermore, the motivational and emotional nature of “engagement” is crucial and differentiates this concept from activation which is rather more cognitive and behavioral in its nature.

Other concepts are strongly anchored to the area of disease management. For instance, the classical concepts of “adherence” and “compliance” express a hierarchical representation of the clinical consultation, where the healthcare professional is considered as “the expert” and he/she prescribes to the patient (not expert) the rules to manage his/her disease. These concepts imply the implicit evaluation of the patient behavior in self-management such as more or less good, more or less able to respond adequately to the expert's requirements (Haynes et al., 1979; Vlasnik et al., 2005). Furthermore, these concepts evoke a medicalizing idea of the patient such as passive and needing to be correct in order to better functioning, not only at the clinical but also at the psychosocial level. It is evident how the concept of “engagement” move from a very different philosophy and representation of the patient role along the healthcare journey. From this perspective, the different actors implied in the healthcare journey are considered “experts” based on their specific subjective experience of illness and of its management. The concept of “engagement” aims at democratizing the clinical consultation and at legitimizing care receivers in a more starring role.

On the other hand, the concepts of “involvement” and “participation,” refers instead to the dyadic context of the medical consultation and the cognitive/emotional attitude of the patient to the negotiation of clinical decision making. There is an evident conceptual link between the concepts of “involvement,” “participation,” and the concept of “engagement.” All of these concepts advocate for the proactive role of patients in the decisions about care. However, these concepts relate to different levels of healthcare services demand-supply exchange (Murray et al., 2006; Thompson, 2007), since the concepts of “involvement” and “participation” are mostly limited to the dyadic context of the doctor-patient consultation in shared decision making, whereas the concept of “engagement,” refers to the role of patients and to how he/she approaches the healthcare system in its complexity (where medical consultation is one of the possible settings).

Finally, the concept of “empowerment” entertains evident areas of overlapping and potential synergies with the concept of “engagement,” although with a different breadth. The concept of “empowerment” relates to the level of patients' power and of agency upon their healthcare condition. Particularly, “empowerment” refers to patients' reacquisition of the subjective sense of control over their disease (Aujoulat et al., 2007). Given this definition, “empowerment” may be defined as a potential prerequisite for the process of engagement, although in turn it is fed by the good experiences that the patient makes in his evolutionary journey of exchange with the healthcare system, and thus along the “patient engagement journey” itself. Furthermore, if “empowerment” is primarily a cognitive and behavioral condition, “engagement” is nurtured by the emotional and motivational components of patients experiences along the care pathway.

Alongside with this desire to engage patients a tendency toward a more patient centered clinical intervention has developed. At the end of the 60 s, Balint introduced the term of patient centered medicine focusing the attention, during the medical encounter, not only to the biological-technical aspects but also on the emotional and relational dimensions (Balint, 1964). The patient-centered medicine is a large concept variously described, till to the proposal of a “transformed clinical method” by the Canadian group: they suggested to involve the patient and consider his/her own perspective (Stewart et al., 2003) not only in the clinical encounter but in the process of care. The patient centered medicine is conceptualized as a clinical method of a bio-psycho-social model of care and has the aim to transform the clinical approach to patients and enhance their involvement. So far, patient engagement and the patient-centered medicine seem to have a common ground and similar aims, but their overlapping is not clear and not frequently explored in the literature. Furthermore, shared guidelines about how to translate into the clinical practice the imperative of patient engagement have still to come. In this light, this Frontiers Research Topics has been conceived as an arena to bridge research and theoretical expertise mastered in different disciplinary domains in order to set the ground for a shared definition of what consumer engagement in health and well-being is and on useful guidelines for practice a consumer engagement in a patient centered medicine.

We invited authors from different disciplinary domains to contribute original as well as review article in order to set a debate about patient engagement applications and its conceptual relationship with patient centered medicine.

In the present issue, readers will find the two concepts differently approached. The importance of engagement in health and well-being increased in the last years outlining the role of patient and legitimizing the starring role in the care receivers is described in the contribution by Bigi: in particular the issue that engagement involves a profound change in the doctor's behavior is discussed. The increase of chronic diseases has highlighted the need for increased engagement in patients (Menichetti and Graffigna Zhang et al.). Menichetti and Graffigna underlined the importance of intervention (PHEinAction) to support patient engagement and lifestyle change and maintenance, facilitating emotional and psychological processes. The relevance of emotion in engagement process appears also in the study conducted on patients with HIV by Leone et al. The importance of this construct in cancer studies (Villani et al. Saita et al.), and mental health studies (Degli Stefani and Biasutti Singh et al. Oliveira-Maia et al.) seems to suggest the usefulness of psychosocial interventions in addressing the care, for patients and the family. Last but not least, the training for health care workers appears to be crucial in order to enable cares to engage patient, particularly for nurses to learn strategies and assessment measures in clinical practice are key points in supporting the realization of patient engagement in healthcare (Barello et al.).

## Author contributions

All authors listed have made a substantial, direct and intellectual contribution to the work, and approved it for publication.

### Conflict of interest statement

The authors declare that the research was conducted in the absence of any commercial or financial relationships that could be construed as a potential conflict of interest. The handling Editor declared a shared affiliation, though no other collaboration, with one of the authors, GG.
